# Circulatory Inflammatory Proteins as Early Diagnostic Biomarkers for Invasive Aspergillosis in Patients with Hematologic Malignancies—an Exploratory Study

**DOI:** 10.1007/s11046-024-00831-8

**Published:** 2024-02-26

**Authors:** Robina Aerts, Isis Ricaño-Ponce, Mariolina Bruno, Toine Mercier, Diletta Rosati, Johan Maertens, Vinod Kumar, Agostinho Carvalho, Mihai G. Netea, Martin Hoenigl, on behalf of the ECMM Immunologic Markers for Treatment Monitoring and Diagnosis in Invasive Mold Infection Working Group Contributors, Rosanne Sprute, Philipp Köhler, Jan Grothe, Cornelia Lass-Flörl, Carol Garcia-Vidal, Patricia Monoz, Jean-Pierre Gangneux, Daniele Giaccobbe, Malgorzata Mikulska

**Affiliations:** 1 Department of Hematology, University Hospitals Leuven, Leuven, Belgium; 2 Department of Microbiology, Immunology and Transplantation, KU Leuven, Herestraat 49, 3000 Leuven, Belgium; 3 Department of Internal Medicine and Radboud Center for Infectious Diseases, Radboud University Medical Centre, Nijmegen, The Netherlands; 4 Life and Health Sciences Research Institute (ICVS), School of Medicine, University of Minho, 4710-057 Braga, Portugal; 5 ICVS/3B’s - PT Government Associate Laboratory, 4806-909 Braga/Guimarães, Portugal; 6 Laboratory of Chemical Biology, Department of Biomedical Engineering and Institute for Complex Molecular Systems, Eindhoven University of Technology, Eindhoven, The Netherlands; 7 Department of Immunology and Metabolism, Life & Medical Sciences Institute, University of Bonn, Bonn, Germany; 8 Biotech Med, Graz, Austria; 9 Translational Medical Mycology Research Unit, ECMM Excellence Center for Medical Mycology, Medical University of Graz, Graz, Austria; 10 Division of Infectious Diseases, Department of Internal Medicine, Medical University of Graz, Auenbruggerplatz 15, 8036 Graz, Austria

**Keywords:** Biomarkers, Proteomics, Invasive fungal infections, Aspergillosis, IL-17, Opportunistic infections

## Abstract

**Objectives:**

Invasive aspergillosis (IA) is a major cause of mortality in immunocompromised patients and it is difficult to diagnose because of the lack of reliable highly sensitive diagnostics. We aimed to identify circulating immunological markers that could be useful for an early diagnosis of IA.

**Methods:**

We collected longitudinally serum samples from 33 cases with probable/proven IA and two matched control cohorts without IA (one with microbiological and clinical evidence of bacterial or viral non-fungal pneumonia and one without evidence of infection, all matched for neutropenia, primary underlying disease, and receipt of corticosteroids/other immunosuppressants) at a tertiary university hospital. In addition, samples from an independent cohort (n = 20 cases of proven/probable IA and 20 matched controls without infection) were obtained. A panel of 92 circulating proteins involved in inflammation was measured by proximity extension assay. A random forest model was used to predict the development of IA using biomarkers measured before diagnosis.

**Results:**

While no significant differences were observed between IA cases and infected controls, concentrations of 30 inflammatory biomarkers were different between cases and non-infected controls, of which nine were independently replicated: PD-L1, MMP-10, Interleukin(IL)-10, IL-15RA, IL-18, IL-18R1, CDCP1, CCL19 and IL-17C. From the differential abundance analysis of serum samples collected more than 10 days before diagnosis and at diagnosis, increased IL-17C concentrations in IA patients were replicated in the independent cohort.

**Conclusions:**

An increased circulating concentration of IL-17C was detected both in the discovery and independent cohort, both at the time of diagnosis and in samples 10 days before the diagnosis of IA, suggesting it should be evaluated further as potential (early) biomarker of infection.

**Supplementary Information:**

The online version contains supplementary material available at 10.1007/s11046-024-00831-8.

## Introduction

Invasive mold infections (IMI) remain associated with high mortality rates, with a 1-year mortality of 32% (even higher in certain populations) [1–3], requiring early diagnosis and prompt initiation of appropriate treatment to increase odds of survival [4]. The early diagnosis of invasive aspergillosis (IA), the most frequent IMI, is a challenge, with signs and symptoms being either unspecific or appearing at a very late disease phase. Histopathological identification of fungal elements within a typical tissue reaction remains the gold-standard method, but the material is rarely accessible, and fungal culture form respiratory specimens has a low sensitivity [5]. Non-culture-based fungal biomarkers such as the detection of galactomannan (GM) or other *Aspergillus* antigens, beta-D-glucan (BDG) and molecular diagnostic tests such as polymerase chain reaction (PCR) and even next generation sequencing are now used as a diagnostic mycological criteria, often in combination [6]. However, their availability varies between geographical regions [7]. In addition, these tests are far from perfect and present several limitations, including the possibility of false positive and false negative results, and the low sensitivity in patients receiving mold-active prophylaxis or treatment [8, 9]. Performance can also vary depending on the tested matrix or on the immunological status of the patients [10]. The predictive value and performance of the currently available biomarkers and tests for early diagnosis of IA may be enhanced by combination with sensitive and specific immunological markers [11].

Cytokines and chemokines which are centrally involved in protective immunity against *Aspergillus* species (spp.) and other molds [12, 13] have been studied singularly or in combination as diagnostic markers for invasive fungal infections [14]. In addition, some studies suggest that increased or decreased concentrations of certain cytokines or chemokines in serum are associated with an heightened risk of IA, so they could be used as a trigger to initiate diagnostic investigation or as potential biomarkers for an early antifungal treatment [15]. Yet, the early predictive potential of immunological markers has not been investigated in detail, and most of the studies often focused on the role of specific cytokines or chemokines leading to bias and missing potentially relevant mechanisms involved.

Therefore, in this study, we set out to systematically explore the early predictive role of immunological serum biomarkers in patients with IA by (i) investigating the proteomic profile of a cohort of individuals not receiving anti-mold prophylaxis, and (ii) using an unbiased approach without pinpointing to preselected cytokines.

## Methods

This retrospective case–control study from the European Conference of Medical Mycology (ECMM) Immunologic Markers for Treatment Monitoring and Diagnosis in Invasive Mold Infection Working Group aimed to identify immunologic markers for early diagnosis of IA in a cohort of hematology patients naïve to mold-active therapy, of which serial serum samples were available.

### Study Design, Participants, and Sample Collection

For the discovery cohort, we retrospectively evaluated longitudinal serum samples from 33 cases with proven or probable IA and 66 matched controls from two matched control cohorts (an infected and a non-infected cohort) without IMI. Patients were classified as per EORTC-MSGERC consensus definitions [6]. Patients with proven or probable IA were selected as cases. High-risk hematology patients (with underlying hematological malignancy, of which some underwent an allogeneic hematopoietic cell transplantation) that underwent a diagnostic workup for clinically suspected invasive fungal infection, but never received anti-mold therapy due to lack of supportive evidence but had microbiological and clinical evidence of bacterial or viral non-fungal infection were considered as “infected controls”. High-risk hematology patients without evidence of infection and without fever were considered as “non-infected controls”.

We also obtained samples from an independent cohort of 20 cases of proven or probable IA and 20 matched controls without evidence of infection, all with underlying hematological malignancies.

All controls were matched in terms of presence of neutropenia, primary underlying disease, and treatment with corticosteroids and/or other immunosuppressants within a week of inclusion. Prior to the diagnosis, none of the patients received anti-mold active prophylaxis.

Samples were collected between April 2017 and April 2021 at the Belgian National Reference Centre for Mycosis (University Hospitals Leuven, Leuven, Belgium). More details on the cohort and a flux diagram of the sample collection is provided in the supplementary material (Online Supplement and Supplementary Fig. 1).

For the cases, samples were selected as close as possible to the date of the test that confirmed diagnosis: *e.g.,* date of positive culture, date of confirming *Aspergillus* PCR, or date of serum or BAL fluid GM that triggered the start of treatment, called the “diagnostic sample” (Timepoint 3). In addition, we selected two serum samples obtained more than 10 days before (Timepoint 1) and two serum samples obtained within 10 days (Timepoint 2) before this diagnostic sample. For the “infected controls”, the sample that was drawn on the day that the patient had one of the following events (in descending order): a bronchoscopy, a high-resolution CT scan of the chest or a fever greater than 38.2 °C, was selected as day 0, and two serum samples in time before this day 0. For the “non-infected controls”, serial samples were selected from an at-random 4-week period during their immunocompromised timeframe within 6 months after the start of an intensive chemotherapy schedule or a hematopoietic stem cell transplantation.

This prospective, noninterventional, systematic sampling was approved by the institutional ethics committee under the number S61797. All patients provided written informed consent before inclusion.

### Proteomic Analysis

Samples were analyzed at the Radboud University Medical Centre, Nijmegen, The Netherlands. A panel of 92 inflammatory markers was measured in serum samples using proximity extension assay (Olink Proteomics AB, Uppsala, Sweden). The OLINK Inflammation Panel includes proteins that are crucial for anti-fungal host immune response: Interleukin(IL)-4, IL-6, IL-8, IL-10, IL-15, IL-17A, IL-22, IL-1β, IL-1 receptor antagonist (IL-1Ra), soluble IL-2 receptor (sIL-2r), TNF, IFN γ, and RANTES (chemokine ligand 5) (for the complete list: https://www.olink.com/products/target/inflammation/) through a Proceek Multiplex proximity extension assay [16]. In this assay, the target proteins are recognized by antibody pairs coupled to cDNA strands. As soon as those probes come in proximity with the target protein the binding happens, enabling the probes to anneal and amplify during real-time polymerase reaction.

Internal controls were used to minimize variation within runs. Detected proteins were normalized and measured on a log2-scale as normalized protein expression value (NPX). The NPX value is different for each protein due to the sensitivity of each of the probes, with range and estimated inversion from NPX value to absolute amount (ng/μL) available on the Olink website (https://olink.com/faq/what-is-npx/). As part of the quality control, proteins detected in less than 75% of the samples and samples that deviated more than 0.3 NPX from the median were removed. Protein concentrations under the detection threshold were replaced with the protein’s lower limit of detection (LOD). NPX values were compared between cases and controls at three different time points: more than 10 days before diagnosis, less than 10 days before diagnosis and at the day of diagnosis.

### Statistical Analysis

Protein levels of cases and controls were compared using a linear regression model using the Limma package [17] in R. Correction for multiple testing was applied by the Benjamini–Hochberg method, and a false discovery rate (FDR) < 0.05 was considered statistically significant. Based on the significant proteins, a random forest model was created in R using the pROC package and the AUC of the model was calculated. Wald and ANNOVA test were performed using the nparLD package in R using the f1.ld.f1 function [18]. In addition to these tests, we also tested a model with the hypothesis of no simple time effects. These analyses were restricted to the 48 individuals for which we had measurements for the three time points. All analyses were performed in R (version 4.0.2, R Foundation for Statistical Computing, Vienna, Austria).

## Results

### Demographics

Clinical and microbiological information on the 33 patients from the discovery cohort and the 20 patients from the independent cohort can be found in Supplementary Table 1. A summary of demographic characteristics of cases and control cohorts is reported in Supplementary Table 2. The independent cohort was associated with significantly higher rates of neutropenia (a neutrophil count < 500/µL for ≥ 10 days at time of diagnosis our temporally related) (85% versus 35%), systemic corticosteroid use (43% vs 12%), active GVHD (15% versus 2%), but lower rates of T-cell suppressive treatments (20% vs 38%) in compared to the discovery cohort (Supplementary Table 2).

**Fig. 1:**
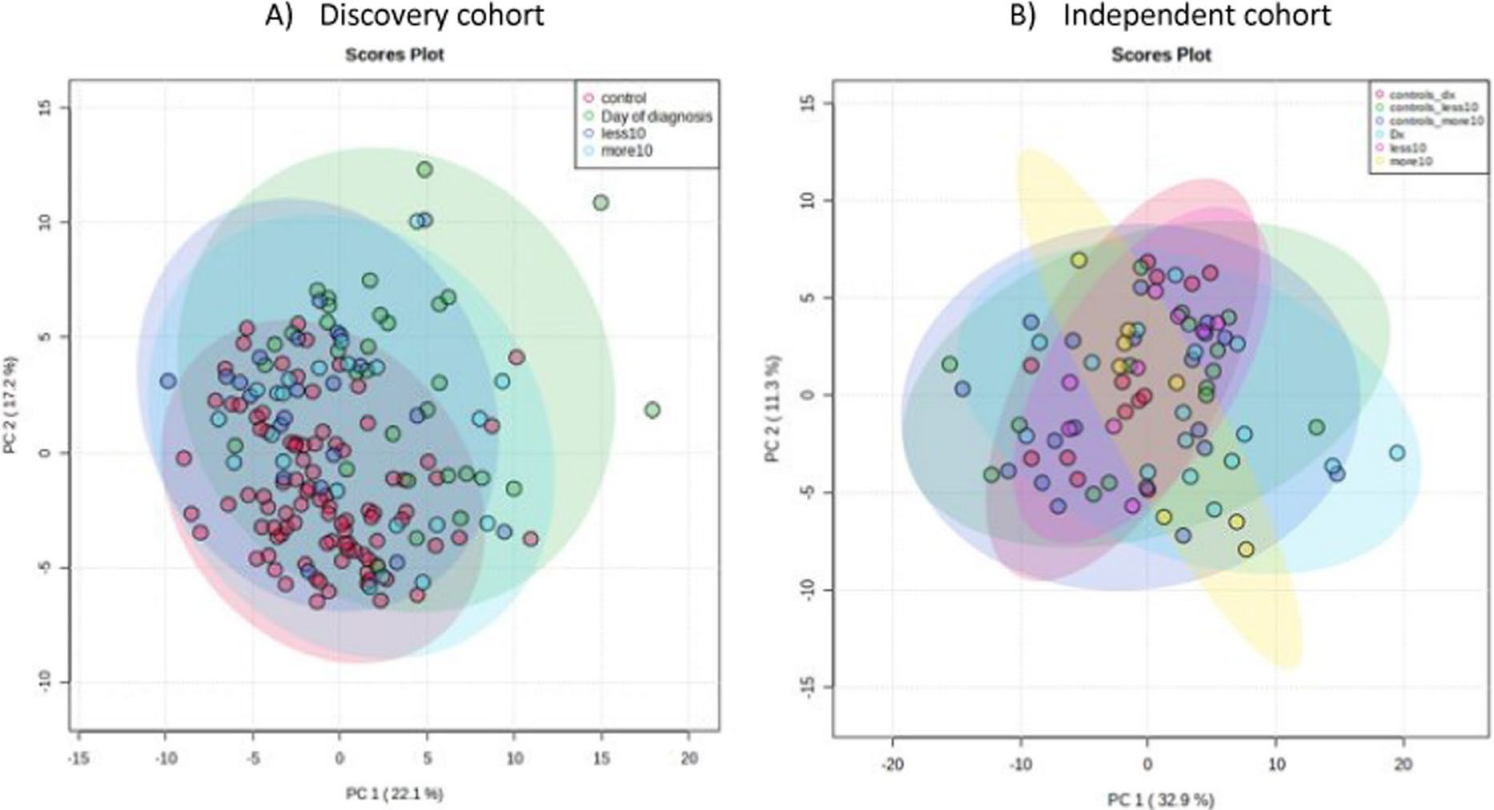
Overview of the stratification of the samples into three groups: samples obtained (a) more than 10 days before the diagnosis of IMI, (b) less than 10 days before diagnosis, and (c) at the time of diagnosis (± 1 day) (Fig. 1A: stratification of the discovery cohort, Fig. 1B: stratification of the independent cohort)

To focus on early diagnosis, samples were stratified into three groups: samples obtained (a) more than 10 days before the diagnosis of IMI, (b) less than 10 days before diagnosis, and (c) at the time of diagnosis (± 1 day) (Fig. 1A; stratification of the validation cohort Fig. 1B).

### Discovery Cohort

#### IA Cases Versus Non Infected Controls

**Fig. 2:**
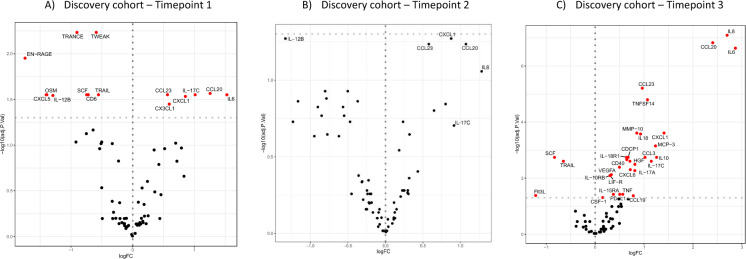
Discovery cohort: **A**Timepoint 1: Abundance analysis more than 10 days before diagnosis comparing cases with non-infected controls. Proteins with significant different concentrations are in red. Six proteins showed significantly higher concentrations: IL-6, CCL20, IL-17C, CXCL1, CCL23, and CX3CL1. Nine proteins had lower concentrations: TRANCE, TWEAK, EN-RAGE, TRAIL, SCF, CD6, IL-12B, OSM, and CXCL5. **B** Timepoint 2: Abundance analysis in serum from samples less than 10 days before diagnosis comparing cases with non-infected controls. No significant differences between cases and controls were found (dots in black not significant). **C** Timepoint 3: Abundance analysis at time of diagnosis comparing cases with non infected controls. 30 proteins were expressed significantly differently. There was a higher concentration of IL-8, CCL20, IL-6, CCL23, TNFS14, CXCL1, MMP-10, IL-18, MCP-3, CDCP1, CCL3, IL-18R1, IL-10, HGF, CD40, ADA, IL-17C, VEGFA, CXCL6, IL-17A, IL-10RB, LIF-R, IL-15RA, TNF, CSF-1, PD-L1, and CCL19, and a lower concentration of SCF, TRAIL, and Tlt3L

In the differential abundance analysis of serum samples collected more than 10 days before diagnosis (timepoint 1), six proteins showed significantly higher concentrations in the cases group compared to the non-infected controls: IL-6, CCL20, IL-17C, CXCL1, CCL23, and CX3CL1. Nine proteins had lower concentrations: TRANCE, TWEAK, EN-RAGE, TRAIL, SCF, CD6, IL-12B, OSM, and CXCL5 (Fig. 2A). We did not observe any proteins significantly different between cases and non-infected controls in the group with less than 10 days before diagnosis (timepoint 2) after correcting for multiple testing (Fig. 2B). Abundance analysis on the day of diagnosis (timepoint 3) revealed 30 differentially regulated proteins, with a higher concentration of IL-8, CCL20, IL-6, CCL23, TNFS14, CXCL1, MMP-10, IL-18, MCP-3, CDCP1, CCL3, IL-18R1, IL-10, HGF, CD40, ADA, IL-17C, VEGFA, CXCL6, IL-17A, IL-10RB, LIF-R, IL-15RA, TNF, CSF-1, PD-L1, and CCL19, and a lower expression of SCF, TRAIL, and Tlt3L (Fig. 2C).

**Fig. 3:**
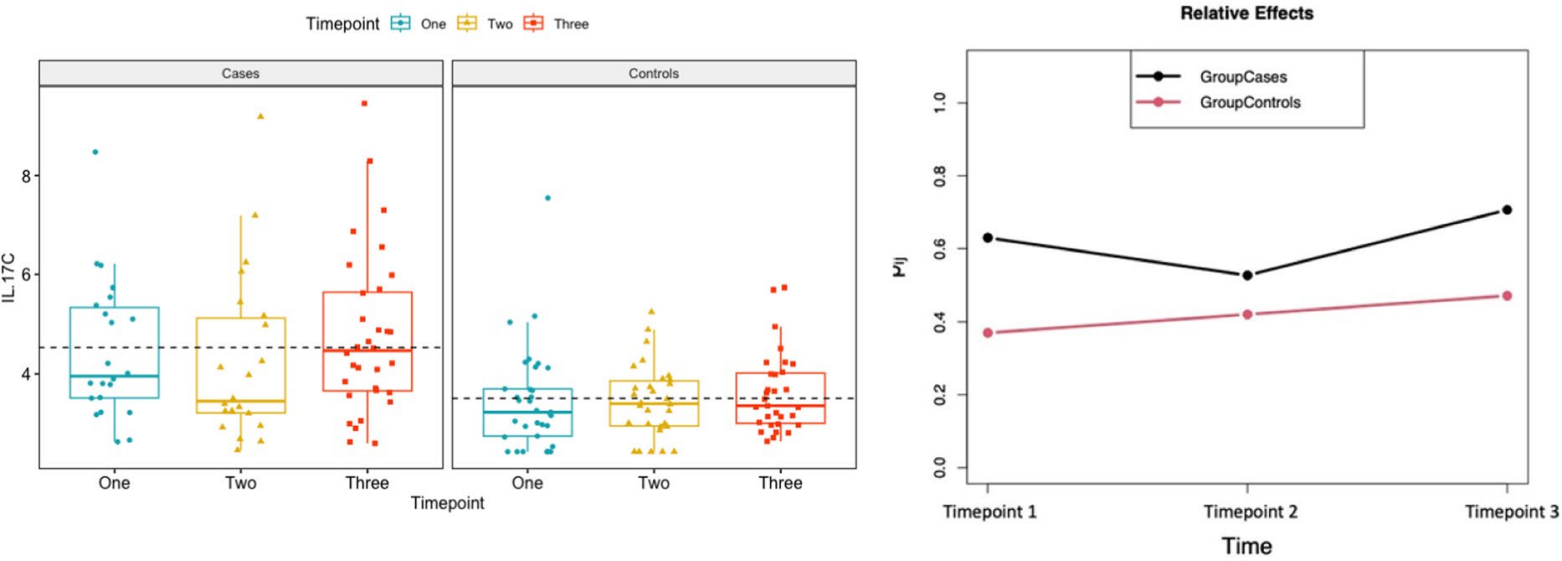
Trajectories over time of IL-17C in cases and controls. Timepoint 1: more than 10 days before the diagnosis of IMI, Timepoint 2: less than 10 days before diagnosis and Timepoint 3: at the time of diagnosis (± 1 day)

The concentrations of IL-17C were increased in the case group at all three time points compared to the non-infected controls. In longitudinal analysis, we observed a general difference in the global patterns of IL-17C between cases and non-infected controls (*p*-value = 0.0018 for Wald and ANOVA tests) and over time (*p*-value = 0.03 for Wald and *p*-value = 0.022 for ANOVA). Additionally, we observed significant differences over time within the case group (*p*-value = 0.04 and *p*-value = 0.025), but not within the non-infected control group (*p*-value = 0.24 and *p*-value = 0.21) (Fig. 3).

**Fig. 4:**
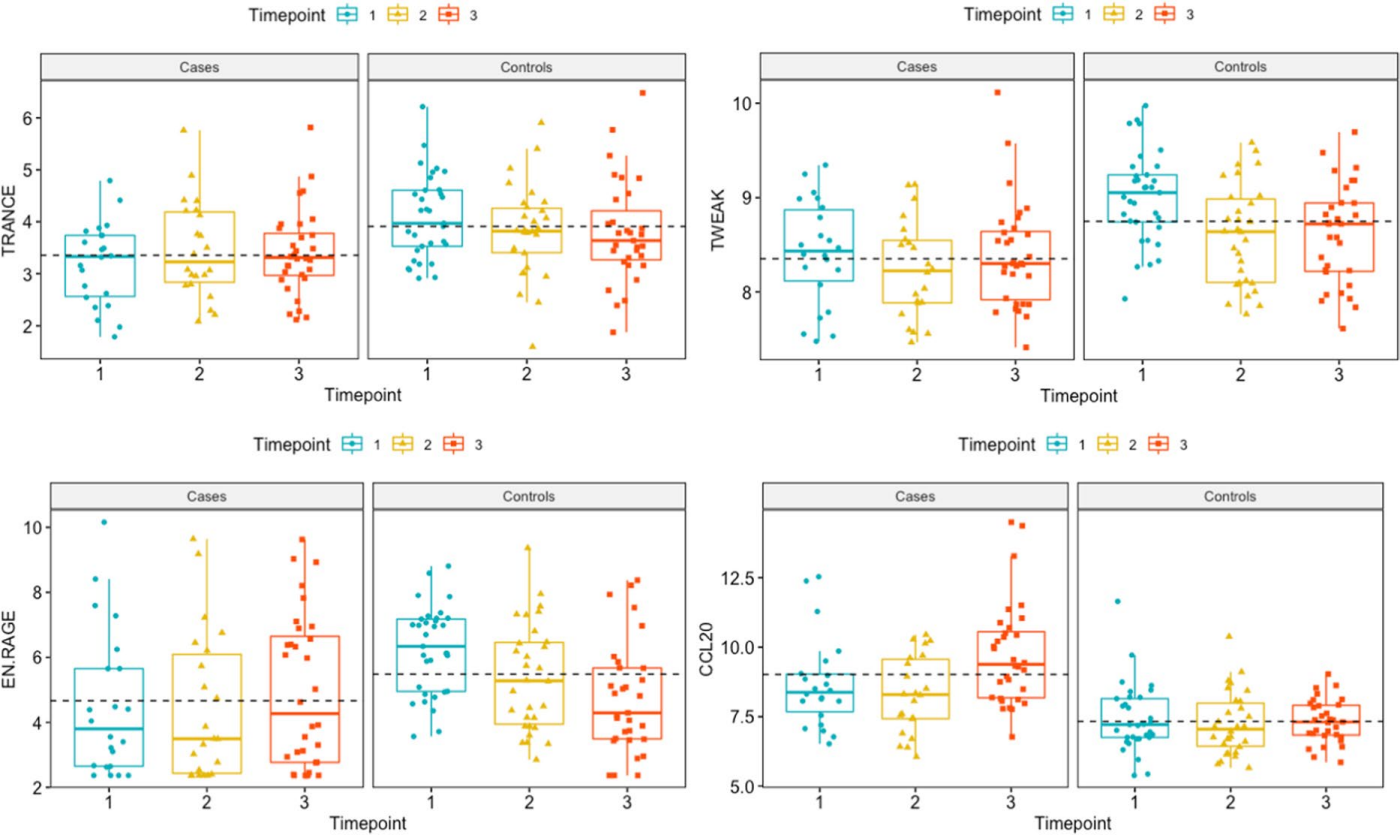
Trajectory of the four most deviant proteins from the discovery cohort TRANCE, TWEAK, EN-RAGE, and CCL20 over time. Timepoint 1: more than 10 days before the diagnosis of IMI, Timepoint 2: less than 10 days before diagnosis and Timepoint 3: at the time of diagnosis (± 1 day)

We then used the samples from cases and non-infected controls collected more than 10 days before diagnosis to create a machine learning algorithm including the four most deviant proteins from the primary cohort: TRANCE, TWEAK, EN-RAGE, and CCL20. The model was able to classify patients later diagnosed with IMI with 87% accuracy (reporting an error rate of 12.73%) (Supplementary Fig. 2). The protein trajectory over time of these four proteins can be found in Fig. 4.

#### IA Cases Versus Infected Controls

When correcting for multiple testing, the comparison between IA cases and infected controls showed no significant differences at any timepoint, including more than 10 days before diagnosis (Supplementary Fig. 3). Without correction for multiple testing, we found some proteins with nominal significant *p*-value: three for samples taken more than 10 days before diagnosis (TRANCE, ADA, and CST5), four for less than 10 days before diagnosis (CXCL10, IL-12B, HGF, and TNFB), and one at the time of diagnosis (IL-10RB).

### Independent Cohort

**Fig. 5:**
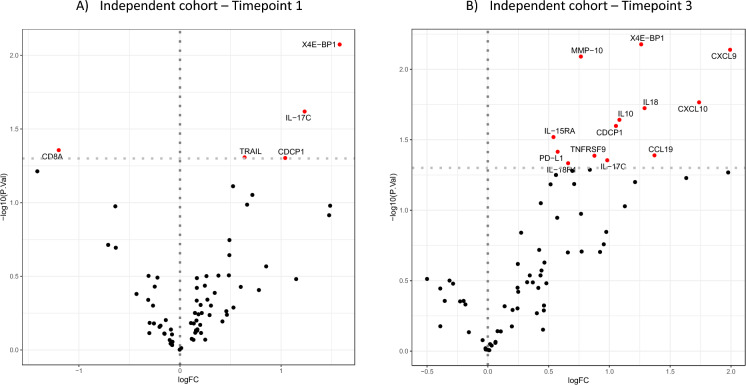
Independent cohort: **A** Timepoint 1: Abundance analysis more than 10 days before diagnosis comparing cases with non-infected controls from the independent cohort. **B **Timepoint 3: Abundance analysis at time of diagnosis comparing cases with non-infected controls from the independent cohort

We performed targeted testing to validate the findings from the discovery cohort in the independent cohort and we found that the increased abundance of IL-17C was replicated with a *p*-value = 0.024 in samples obtained more than 10 days before diagnosis (timepoint 1) (Fig. 5A). Out of 30 differentially regulated proteins found at time of diagnosis (timepoint 3) in the discovery cohort, nine were also replicated in this independent cohort: PD-L1, MMP-10, IL-10, IL-15RA, IL-18, IL-18R1, CDCP1, CCL19, and IL-17C being more abundant among the cases (unadjusted *p*-value < 0.05; adjusted *p*-value > 0.5) (Fig. 5B). The derived machine learning model could not be replicated in the independent cohort, as the accuracy for this cohort was 47% (error rate of 53%).

## Discussion

We systematically investigated the diagnostic role of immunological serum biomarkers in IA and found significant differences in 30 inflammatory proteins between patients with or without IA at the time of diagnosis, of which PD-L1, MMP-10, IL-10, IL-15RA, IL-18, IL-18R1, CDCP1, CCL19, and IL-17C were replicated in the independent cohort at the same timepoint. IL-17C was the only protein with a higher concentration at 10 days before the diagnosis of IA which was replicated in the independent cohort at the same timepoint. IL-17C therefore was the only “biomarker” that showed increased concentrations at both timepoints in both cohorts when comparing cases to non-infected controls.

Several layers of host barriers are responsible for the protection against the hundreds to thousands of conidia inhaled every day by humans [19] resulting in elevated levels of cytokines (details outlined in the online supplement). In our study, a higher circulating concentration of IL-17C was observed both in the discovery and the validation cohort, both at the time of diagnosis and in samples 10 days before the diagnosis of IA. IL-17C shares 27% amino acid homology with IL-17A, which is produced mainly by leukocytes (Th4 cells and neutrophils) and is crucial for a protective mucosal anti-fungal host response, with elevated levels in IMI reported before [20–24]. IL-17C specifically binds to the receptor IL-17RA/RE, a complex that is expressed on both epithelial and Th17 cells [25]. Several cell types, including lung epithelial cells, induce the production of IL-17C, which in turn, is responsible for the induction of the pro-inflammatory response at the site of the epithelial barrier and antibacterial functions [26]. IL-17C production activates expression of other proinflammatory cytokines and of chemokines such as CXCL1/2/3 and CCL20, which were also elevated in our cohort at time of diagnosis and 10 days before diagnosis [26]. It has been shown to enhance the expression of cytokines and chemokines in epithelial cells in an autocrine manner, resulting in increased pulmonary inflammation [27]. The role of IL-17C in IA or other mold infections remains still unknown.

IL-17C not only induces inflammation, but also promotes tissue healing. For instance, during oral, skin, and bloodstream candidiasis, mice lacking IL-17C are still able to clear *C. albicans* suggesting a dispensable protective role for this cytokine [28]. However, in another study, mice that lack IL-17C displayed an increased survival and attenuated kidney damage, although the fungal load was similar to the wild type, thus suggesting an immunopathological role for IL-17C during systemic *Candida* infection [29]. IL-17C was also involved in the immune response of respiratory epithelial cells: IL-17C heightened inflammatory responses of respiratory epithelial cells infected with *Pseudomonas aeruginosa* and was found in bronchial tissue of subjects with infection-related lung diseases [30]. Moreover, IL-17C exacerbates chronic obstructive pulmonary disease (COPD) through neutrophil recruitment via upregulation of CXCL1 [31]. CXCL1 expression was also higher in cases versus non-infected controls in our discovery cohort, significant at timepoint 1 and 3, but non-significant at timepoint 2.

To the best of our knowledge, our study is the first to mention a possible role of IL-17C in invasive mold infection: specifically, these data sets IL-17C as an attractive candidate for early biomarkers for detecting IMI already 10 days before the diagnosis. Moreover, this “early” feature of IL-17C that we found is not surprising, as it is expressed by epithelial cells during bacterial infections at earlier timepoints in disease than IL-17A [27, 32]. Therefore, the fact that IL-17C is already upregulated 10 days before patients develop IMI should not be seen as protective factor, since this cytokine might generate an autocrine loop of autoinflammation driving immunopathology. Further studies that specifically investigate the role of IL-17C on mold infections are warranted to pinpoint its pathophysiologic role during invasive fungal infections.

We also found that PD-L1 concentration was increased at time of diagnosis in both cohorts. The checkpoint protein PD-L1 is best known in the field of immunotherapy where immune checkpoint inhibitors are used for treatment of various cancer types. PD-L1 can be stimulated by IFN-γ and is expressed on macrophages. It promotes T-cell apoptosis, anergy and functional exhaustion [33]. Because immune checkpoint inhibitors restore T-cell response, they could also be active against fungal infections [34]. Previous studies showed that during *C. albicans* infection, PD-L1 is induced by presence of glucans and governs the mobilization of neutrophils through regulating their secretion of CXCL1 and CXCL2 (in this study CXCL1 concentration was also higher in the case group). PD-L1 expression in infected mice strongly correlated with fungal tissue burden and PD-L1 deficiency improved survival, suggesting a negative role of PD-L1 expression on the host immune response inhibiting neutrophil migration from the bone marrow into the infected sites [35, 36]. In mice with mucormycosis and in mice infected with *Histoplasma capsulatum*, inhibition of the PD-L1 pathway has improved clinical outcomes [37–39]. Information specifically for aspergillosis is, however, lacking. These literature findings suggest that PD-L1, instead of being a diagnostic biomarker, could be a marker for treatment decisions. As all high-risk patients included in our cohort had underlying haematological malignancies, knowing more about the possible anti-tumour and anti-fungal synergy of immune checkpoint inhibitors in these patients and its effect on the PD-L1 expression in their serum and BALf would be very interesting. Probably, more information on this will come in the future as trials with immune checkpoint inhibitors and hypomethylating drugs in in patients with AML and MDS are ongoing [34].

We also found that IL-10, IL-15RA, IL-18, and IL-18R1 concentrations were higher at the time of diagnosis in both cohorts. Increased concentrations of these interleukins have been described previously in mice and in patients with IA [13, 40–42]. There was no difference between cases and controls in the days before diagnosis, limiting their potential in early diagnosis or screening. Matrix metalloproteinase (MMP)-10, CCL19, and CDCP1 were found in higher concentrations at time of diagnosis in both the discovery and the independent cohort. However, little is known about their role in mold infections. MMPs play a role in regulating the immune response in inflammatory processes and regarding fungal infections this has been described in host immunity to pulmonary *Cryptococcus* infection [43, 44]. CCL19 plays a role in attracting immune cells to the site of inflammation or infection in general, and this has been shown in the presence of *A. fumigatus* as well [45, 46]. Less information specifically directed towards (fungal) infections is available for CDCP1 but it has for example been described in association with severe COVID-19 infection [47]. As concentrations of these proteins were not significantly different between uninfected and infected patients, and they are described in literature in many other types of infections (and even in autoimmune disease), one could suggest that these proteins are just markers of general immune activation.

As expected, in our discovery cohort, also IL-6 and IL-8 concentrations were higher in serum of cases than in non-infected controls at the time of diagnosis (IL-6 expression also at more than 10-days before diagnosis) as they are well known to be produced by alveolar macrophages and lung epithelial cells in elevated levels in serum and BALf of patients with IMI [11], which has been studied in vitro [48–50] and in clinical case–control studies [13, 20, 51]. Cytokines such as IL-6 and IL-8 have also been shown to be useful in treatment monitoring and outcome prediction of patients with IMI [52, 53]. However, in our study, a higher concentration of IL-6 and IL-8 was not replicated in the independent cohort. Reasons for these divergent findings could include differences in clinical characteristics, with the independent cohort showing a significantly higher rate of neutropenia compared to the discovery cohort, which may impact levels of these inflammatory markers. IL-6 and IL-8 were not significantly higher in serum samples around 10 days before the diagnosis of IMI, limiting their role in screening.

In this study we also identified a model to predict IMI more than 10 days before diagnosis in the discovery cohort. However, the model could not be replicated in the independent cohort, likely due to different clinical characteristics and a small sample size resulting in an underpowered independent cohort.

The major limitation of this study is the relatively small sample size, which is often the case in studies concerning IMI. It is also important to note the lack of significant differences between cases and infected controls, which could be due to the limitations discussed above (including the small sample size). On the other hand, this could also mean that the higher protein concentrations that we found in the comparison between cases and non-infected controls suggest a signal for infection and not specifically a signal for IMI, that could mainly trigger further diagnostic testing. Importantly not only fungal, but also bacterial, viral, and parasitic coinfections including mixed infections are a major threat in patients with underlying haematological malignancies, whereby there are important interactions, e.g. with bacterial or viral infections creating a favorable environment for fungal infections [54–56]. Even though being less specific for one particular causative pathogen, these early biomarkers could still have an important role in stratifying diagnostic testing in these high-risk patients, and in decisions upon prophylactic or preemptive therapy. Other limitations confer to the validation cohort, which was recruited at the same center as the derivation cohort and even more importantly significantly differed in a number underlying disease characteristics. Future studies in independent cohorts with clinical characteristics more similar to our discovery cohort are needed to further validate our findings and particularly the derived model.

## Conclusion

Our results suggest significant differences in the inflammatory proteins between patients with IA and matched controls without infection at the time of diagnosis, but the prediction capacity significantly decreases in earlier samples taken before the diagnosis of the infection. We found in both our derivation and validation cohort that IL-17C may have potential to be a biomarker for early detection of IA; future studies should confirm our findings before the biomarker can be integrated into clinical routine. Future studies are also needed to validate our predictive model in at-risk patients and to evaluate the potential of inflammatory proteins for treatment stratification and outcome prediction in patients with IA.

### Supplementary Information

Below is the link to the electronic supplementary material.

Supplementary file1 (DOCX 888 KB)

## References

[1] Hoenigl M, Salmanton-García J, Walsh TJ (2021). Global guideline for the diagnosis and management of rare mould infections: an initiative of the European confederation of medical mycology in cooperation with the international society for human and animal mycology and the American society for microbiology. Lancet Infect Dis.

[2] Hoenigl M, Seidel D, Carvalho A (2022). The emergence of COVID-19 associated mucormycosis: a review of cases from 18 countries. Lancet Microbe.

[3] Henao-Martínez AF, Corbisiero MF, Salter I, Chastain DB, Thompson GR (2023). Invasive pulmonary aspergillosis real-world outcomes: Clinical features and risk factors associated with increased mortality. Med Mycol.

[4] Cornely OA, Lass-Flörl C, Lagrou K, Arsic-Arsenijevic V, Hoenigl M (2017). Improving outcome of fungal diseases—guiding experts and patients towards excellence. Mycoses.

[5] Hoenigl M, Prattes J, Spiess B (2014). Performance of galactomannan, beta-d-glucan, Aspergillus lateral-flow device, conventional culture, and PCR tests with bronchoalveolar lavage fluid for diagnosis of invasive pulmonary aspergillosis. J Clin Microbiol.

[6] Donnelly JP, Chen SC, Kauffman CA (2020). Revision and update of the consensus definitions of invasive fungal disease from the european organization for research and treatment of cancer and the mycoses study group education and research consortium. Clin Infect Dis.

[7] Salmanton-García J, Hoenigl M, Gangneux J-P (2023). The current state of laboratory mycology and access to antifungal treatment in Europe: a European confederation of medical mycology survey. The Lancet Microbe.

[8] Eigl S, Prattes J, Reinwald M (2015). Influence of mould-active antifungal treatment on the performance of the Aspergillus-specific bronchoalveolar lavage fluid lateral-flow device test. Int J Antimicrob Agents.

[9] Springer J, Lackner M, Nachbaur D (2016). Prospective multicentre PCR-based Aspergillus DNA screening in high-risk patients with and without primary antifungal mould prophylaxis. Clin Microbiol Infect.

[10] Bassetti M, Peghin M, Vena A (2018). Challenges and solution of invasive aspergillosis in non-neutropenic patients: a review. Infect Dis Ther.

[11] Jenks JD, Rawlings SA, Garcia-Vidal C (2019). Immune parameters for diagnosis and treatment monitoring in invasive mold infection. J Fungi (Basel).

[12] Winn RM, Gil-Lamaignere C, Roilides E (2003). Selective effects of interleukin (IL)-15 on antifungal activity and IL-8 release by polymorphonuclear leukocytes in response to hyphae of aspergillus species. J Infect Dis.

[13] Heldt S, Eigl S, Prattes J (2017). Levels of interleukin (IL)-6 and IL-8 are elevated in serum and bronchoalveolar lavage fluid of haematological patients with invasive pulmonary aspergillosis. Mycoses.

[14] Wang Q, Yang M, Wang C, Cui J, Li X, Wang C (2020). Diagnostic efficacy of serum cytokines and chemokines in fungal bloodstream infection in febrile patients. J Clin Lab Anal.

[15] Thammasit P, Sripetchwandee J, Nosanchuk JD, Chattipakorn SC, Chattipakorn N, Youngchim S (2021). Cytokine and chemokine responses in invasive aspergillosis following hematopoietic stem cell transplantation: past evidence for future therapy of aspergillosis. J Fungi..

[16] Assarsson E, Lundberg M, Holmquist G (2014). Homogenous 96-plex PEA immunoassay exhibiting high sensitivity, specificity, and excellent scalability. PLoS ONE.

[17] Ritchie ME, Phipson B, Wu D (2015). limma powers differential expression analyses for RNA-sequencing and microarray studies. Nucleic Acids Res.

[18] Noguchi K, Gel Y, Brunner E, Konietschke F. nparLD: an R software package for the nonparametric analysis of longitudinal data in factorial experiments. Journal of Statistical Software [Internet]. **2012**; 50(12). Available from: https://www.jstatsoft.org/v50/i12/

[19] Tischler BY, Hohl TM (2019). Menacing mold: recent advances in Aspergillus pathogenesis and host defense. J Mol Biol.

[20] Gonçalves SM, Lagrou K, Rodrigues CS, et al. Evaluation of bronchoalveolar lavage fluid cytokines as biomarkers for invasive pulmonary aspergillosis in at-risk patients. Frontiers in Microbiology [Internet]. **2017** [cited 2023 Feb 3]; [8]. Available from: https://www.frontiersin.org/articles/10.3389/fmicb.2017.02362 10.3389/fmicb.2017.02362 PMC5712575 29238334

[21] Zelante T, De Luca A, Bonifazi P (2007). IL-23 and the Th17 pathway promote inflammation and impair antifungal immune resistance. Eur J Immunol.

[22] Becker KL, Ifrim DC, Quintin J, Netea MG, van de Veerdonk FL (2015). Antifungal innate immunity: recognition and inflammatory networks. Semin Immunopathol.

[23] Li H, Chen J, Huang A (2000). Cloning and characterization of IL-17B and IL-17C, two new members of the IL-17 cytokine family. Proc Natl Acad Sci.

[24] Nies JF, Panzer U. IL-17C/IL-17RE: emergence of a unique axis in TH17 biology. Frontiers in Immunology [Internet]. **2020** [cited 2023 Mar 7]; 11. Available from: https://www.frontiersin.org/articles/10.3389/fimmu.2020.00341 10.3389/fimmu.2020.00341 PMC7054382 32174926

[25] Chang SH, Reynolds JM, Pappu BP, Chen G, Martinez GJ, Dong C (2011). Interleukin-17C promotes Th17 cell responses and autoimmune disease via interleukin-17 receptor E. Immunity.

[26] Brevi A, Cogrossi LL, Grazia G, et al. Much more than IL-17A: cytokines of the IL-17 family between microbiota and cancer. frontiers in immunology [Internet]. **2020** [cited 2023 Feb 7]; 11. Available from: https://www.frontiersin.org/articles/10.3389/fimmu.2020.565470 10.3389/fimmu.2020.565470 PMC7683804 33244315

[27] Ramirez-Carrozzi V, Sambandam A, Luis E (2011). IL-17C regulates the innate immune function of epithelial cells in an autocrine manner. Nat Immunol.

[28] Conti HR, Whibley N, Coleman BM, Garg AV, Jaycox JR, Gaffen SL (2015). Signaling through IL-17C/IL-17RE is dispensable for immunity to systemic, oral and cutaneous candidiasis. PLoS ONE.

[29] Huang J, Meng S, Hong S, Lin X, Jin W, Dong C (2016). IL-17C is required for lethal inflammation during systemic fungal infection. Cell Mol Immunol.

[30] Pfeifer P, Voss M, Wonnenberg B (2013). IL-17C Is a mediator of respiratory epithelial innate immune response. Am J Respir Cell Mol Biol.

[31] Jamieson KC, Traves SL, Kooi C (2019). Rhinovirus and bacteria synergistically induce IL-17C release from human airway epithelial cells to promote neutrophil recruitment. J Immunol.

[32] Song X, Zhu S, Shi P (2011). IL-17RE is the functional receptor for IL-17C and mediates mucosal immunity to infection with intestinal pathogens. Nat Immunol.

[33] Butte MJ, Keir ME, Phamduy TB, Sharpe AH, Freeman GJ (2007). Programmed death-1 ligand 1 interacts specifically with the B7–1 costimulatory molecule to inhibit T cell responses. Immunity.

[34] Daver N, Kontoyiannis DP (2017). Checkpoint inhibitors and aspergillosis in AML: the double hit hypothesis. Lancet Oncol.

[35] Yu Y, Wang R-R, Miao N-J (2022). PD-L1 negatively regulates antifungal immunity by inhibiting neutrophil release from bone marrow. Nat Commun.

[36] Wurster S, Albert ND, Kontoyiannis DP (2022). Candida auris bloodstream infection induces upregulation of the PD-1/PD-L1 immune checkpoint pathway in an immunocompetent mouse model. Msphere..

[37] Grimaldi D, Pradier O, Hotchkiss RS, Vincent J-L (2017). Nivolumab plus interferon-γ in the treatment of intractable mucormycosis. Lancet Infect Dis.

[38] Wurster S, Albert ND, Bharadwaj U (2022). Blockade of the PD-1/PD-L1 immune checkpoint pathway improves infection outcomes and enhances fungicidal host defense in a murine model of invasive pulmonary mucormycosis. Front Immunol.

[39] Lázár-Molnár E, Gácser A, Freeman GJ, Almo SC, Nathenson SG, Nosanchuk JD (2008). The PD-1/PD-L costimulatory pathway critically affects host resistance to the pathogenic fungus Histoplasma capsulatum. Proc Natl Acad Sci U S A.

[40] Thakur R, Anand R, Tiwari S, Singh AP, Tiwary BN, Shankar J (2015). Cytokines induce effector T-helper cells during invasive aspergillosis; What we have learned about T-helper cells?. Front Microbiol.

[41] Mencacci A, Cenci E, Bacci A, Montagnoli C, Bistoni F, Romani L (2000). Cytokines in candidiasis and aspergillosis. Curr Pharm Biotechnol.

[42] Warris A, Netea MG, Verweij PE (2005). Cytokine responses and regulation of interferon-gamma release by human mononuclear cells to Aspergillus fumigatus and other filamentous fungi. Med Mycol.

[43] Supasorn O, Sringkarin N, Srimanote P, Angkasekwinai P (2016). Matrix metalloproteinases contribute to the regulation of chemokine expression and pulmonary inflammation in Cryptococcus infection. Clin Exp Immunol.

[44] García-López C, Rodríguez-Calvo-de-Mora M, Borroni D, Sánchez-González J-M, Romano V, Rocha-de-Lossada C (2023). The role of matrix metalloproteinases in infectious corneal ulcers. Surv Ophthalmol.

[45] Gafa V, Remoli ME, Giacomini E (2007). In vitro infection of human dendritic cells by aspergillus fumigatus conidia triggers the secretion of chemokines for neutrophil and Th1 lymphocyte recruitment. Microbes Infect.

[46] Hartigan AJ, Westwick J, Jarai G, Hogaboam CM (2009). CCR7 deficiency on dendritic cells enhances fungal clearance in a murine model of pulmonary invasive aspergillosis1. J Immunol.

[47] Blanco J-R, Cobos-Ceballos M-J, Navarro F (2022). Elevated levels of serum CDCP1 in individuals recovering from severe COVID-19 disease. Aging (Albany NY).

[48] Borger P, Koëter GH, Timmerman JA, Vellenga E, Tomee JF, Kauffman HF (1999). Proteases from Aspergillus fumigatus induce interleukin (IL)-6 and IL-8 production in airway epithelial cell lines by transcriptional mechanisms. J Infect Dis.

[49] Bidula S, Sexton DW, Abdolrasouli A (2015). The serum opsonin L-ficolin is detected in lungs of human transplant recipients following fungal infections and modulates inflammation and killing of aspergillus fumigatus. J Infect Dis.

[50] Ghufran MS, Ghosh K, Kanade SR (2017). A fucose specific lectin from Aspergillus flavus induced interleukin-8 expression is mediated by mitogen activated protein kinase p38. Med Mycol.

[51] Heldt S, Prattes J, Eigl S (2018). Diagnosis of invasive aspergillosis in hematological malignancy patients: performance of cytokines, Asp LFD, and Aspergillus PCR in same day blood and bronchoalveolar lavage samples. J Infect.

[52] Chai LA, Netea MG, Teerenstra S (2010). Early proinflammatory cytokines and C-reactive protein trends as predictors of outcome in invasive aspergillosis. J Infect Dis.

[53] Nouér SA, Nucci M, Kumar NS, Grazziutti M, Barlogie B, Anaissie E (2011). Earlier response assessment in invasive aspergillosis based on the kinetics of serum aspergillus galactomannan: proposal for a new definition. Clin Infect Dis.

[54] Geurts K, Zweijpfenning SMH, Pennings LJ, et al. Nontuberculous mycobacterial pulmonary disease and Aspergillus co-infection: Bonnie and Clyde? European Respiratory Journal [Internet]. European Respiratory Society; **2019** [cited 2023 Dec 22]; 54(1). Available from: https://erj-ersjournals-com.kuleuven.e-bronnen.be/content/54/1/1900117 10.1183/13993003.00117-2019 30956203

[55] Hoenigl M, Seidel D, Sprute R (2022). COVID-19-associated fungal infections. Nat Microbiol.

[56] Ni H, Yu H, Lin Q, Zhong J, Sun W, Nie H (2023). Analysis of risk factors of fungal superinfections in viral pneumonia patients: a systematic review and meta-analysis. Immun Inflamm Dis.

[57] Cunha C, Aversa F, Lacerda JF (2014). Genetic Ptx3 deficiency and aspergillosis in stem cell transplantation. New Engl J Med..

[58] Fisher CE, Hohl TM, Fan W (2017). Validation of single nucleotide polymorphisms in invasive aspergillosis following hematopoietic cell transplantation. Blood.

[59] Thompson GR, Young J-AH (2021). Aspergillus infections. New Engl J Med..

[60] Akoumianaki T, Kyrmizi I, Valsecchi I (2016). Aspergillus cell wall melanin blocks LC3-associated phagocytosis to promote pathogenicity. Cell Host Microbe.

[61] Camargo JF, Husain S (2014). Immune correlates of protection in human invasive aspergillosis. Clin Infect Dis.

[62] Leal SM, Roy S, Vareechon C (2013). Targeting iron acquisition blocks infection with the fungal pathogens aspergillus fumigatus and fusarium oxysporum. PLoS Pathog.

[63] Hebart H, Bollinger C, Fisch P (2002). Analysis of T-cell responses to Aspergillus fumigatus antigens in healthy individuals and patients with hematologic malignancies. Blood.

[64] Garcia-Vidal C, Viasus D, Carratalà J (2013). Pathogenesis of invasive fungal infections. Curr Opin Infect Dis.

[65] Dewi IMW, Van de Veerdonk FL, Gresnigt MS (2017). The multifaceted role of T-helper responses in host defense against aspergillus fumigatus. J Fungi..

