# Supplementary material for: Circulatory Inflammatory Proteins as Early Diagnostic Biomarkers for Invasive Aspergillosis in Patients with Hematologic Malignancies—an Exploratory Study
Source: Mycopathologia. 2024 Feb 26;189(2):24. doi: 10.1007/s11046-024-00831-8 (PMC10896822; doi:10.1007/s11046-024-00831-8)
Supplement: Supplementary file 1 — Supplementary file1 (DOCX 888 KB) [file 11046_2024_831_MOESM1_ESM.docx]

**Supplementary material**

Online Supplement

*Supplement to Methods:*

Adult patients with underlying hematological disorders at risk for developing IMI were screened and followed under a preemptive antifungal strategy: all patients received fluconazole (400 mg/day) to prevent Candida infection, while no mold-active prophylaxis nor empirical therapy was given. Instead, patients were screened twice weekly with serum GM. After 96 to 120 h of fever unresponsive to broad-spectrum antibiotics or clinical signs or symptoms suggestive of pulmonary disease or serum GM detection (OD index ≥ 0.5), a computed tomography (CT) scan of the chest was performed. In case of any abnormality, bronchoscopy and collection of bronchoalveolar lavage fluid (BALf) were performed for extensive microbiological testing (including GM detection using a GM index cutoff ≥1.0) and microscopic analysis, when possible and clinically indicated. Serum samples of these patients were collected twice weekly whenever the patient was hospitalized or during outpatient visits. Samples were stored at -80°C for further analyses.

*Supplement to Discussion:*

Several layers of host barriers are responsible for the protection against the hundreds to thousands of conidia inhaled every day by humans [19]. The proximal airways remove fungal conidia through mucociliary clearance. Airway epithelial cells and alveolar macrophages are the first line of defense against potential mold infections by killing phagocytosed conidia. Dectin-1, dendritic-cell-specific intercellular adhesion molecule 3-grabbing non-integrin (DC-SIGN) and pentraxin 3 have been identified as key molecules in the phagocytosis of these conidia, after which NADPH-dependent reactive oxidant species (ROS) cause killing and further activation of intracellular mechanisms, such as LC3-associated phagocytosis (LAP) [57–60]. The lung epithelial cells and macrophages, through different signaling pathways, such as the caspase recruitment domain-containing protein 9 (CARD9) signaling, produce cytokines (such as tumor necrosis factor (TNF), IL-6, IL-1b) and chemokines (such as IL-8, CCL20, CCL23, CXCL1, CX3CL1) causing in return neutrophil recruitment [12,61]. Neutrophils also employ NADPH-oxidase type II mediated killing, together with the release antimicrobial peptides (defensins) and proteases to arrest iron availability for the fungus [62]. The cytokine and chemokine milieu during antigen presentation shapes the anti-*Aspergillus* T-cell (both CD4 and CD8) response that confers protection against fungal disease [63]. Various molds have been shown to induce T-helper cell (Th) 1, Th2, Th9, and Th17 subsets resulting not only in potent anti-*Aspergillus* T-cells effector mechanisms, but also in elevated serum levels of several cytokines such as interferon (IFN)-γ and IL-6, IL-8, IL-15, and IL-17 [61,63,64,40,65].

Supplementary Figure 1

Overview of the serum sample collection.


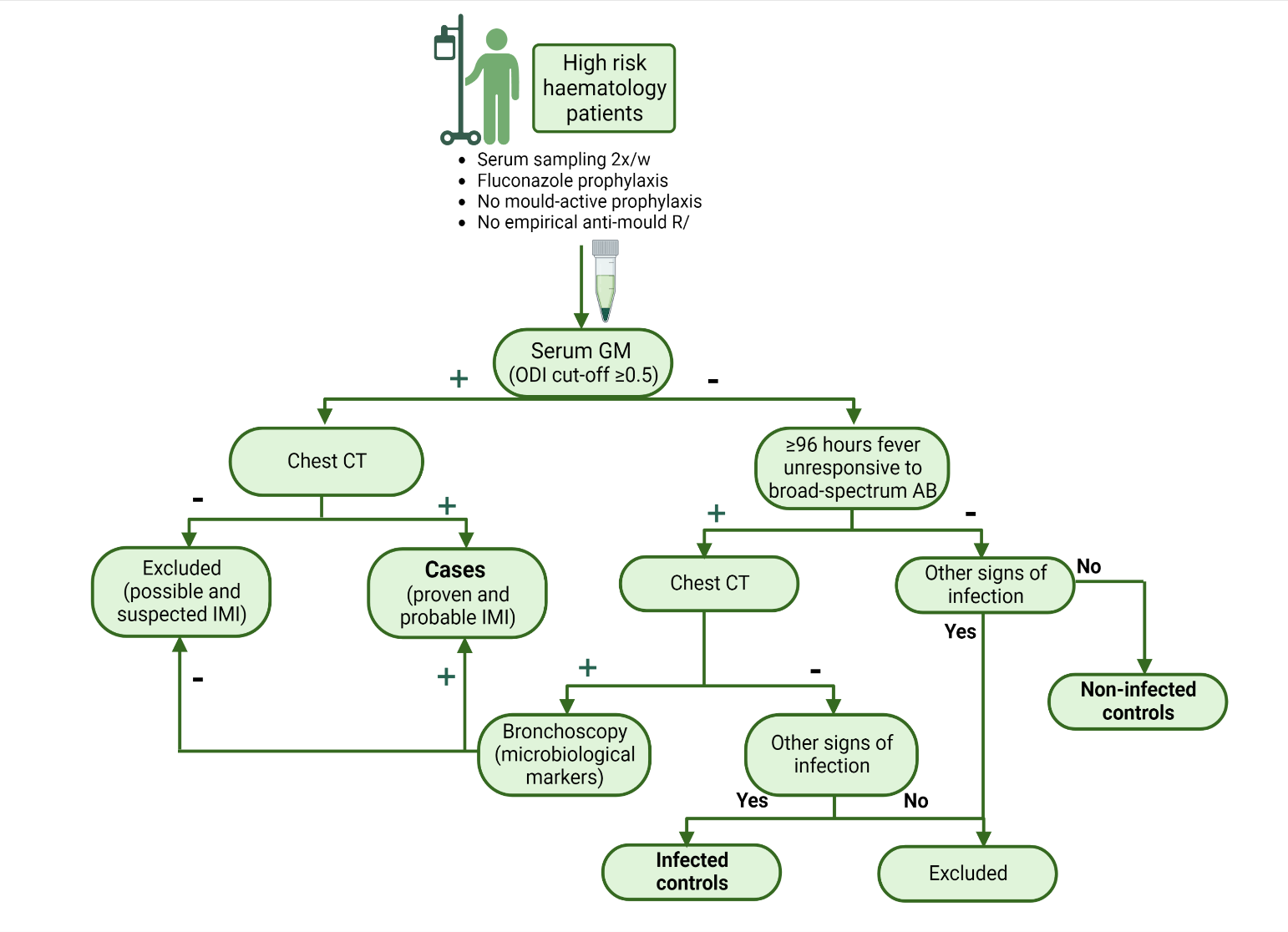


Supplementary Figure 2

Random forest model for the classification of the patients later diagnosed with IMI using the samples collected more than 10 days before diagnosis including the four most deviant proteins from the discovery cohort: TRANCE TWEAK, EN-RAGE, and CCL20 (87% of accuracy, estimated error rate of 12.73%).


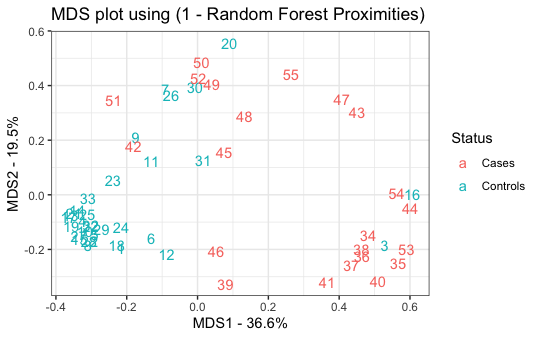


Supplementary Figure 3

Discovery cohort – Timepoint 1: Abundance analysis more than 10 days before diagnosis comparing cases with infected controls. No significant differences between cases and infected controls were found (dots in black not significant).


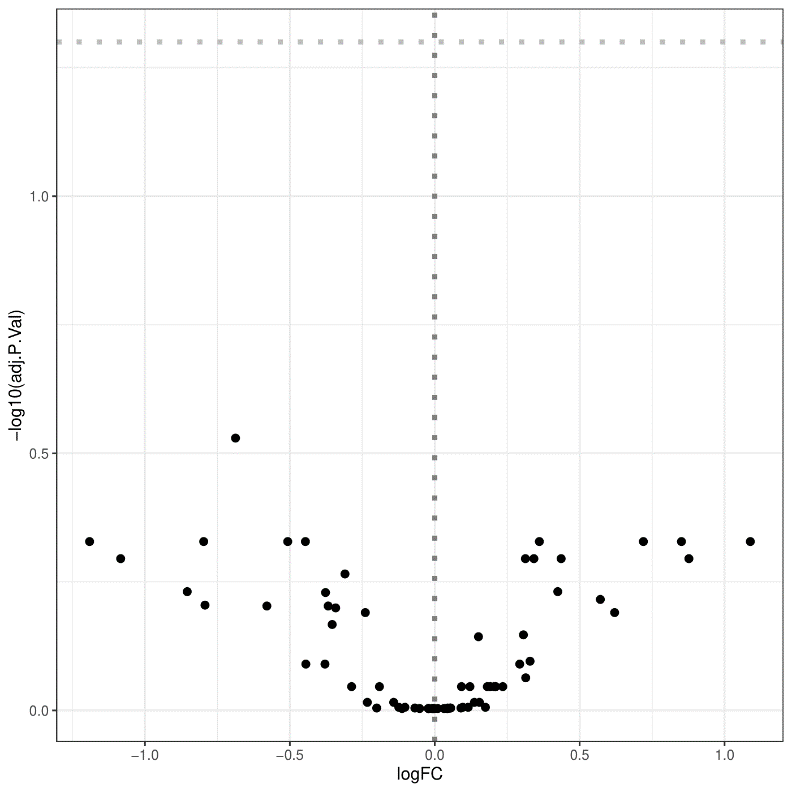


Supplementary Table 1

Clinical and microbiological information of the 33 cases included in the discovery cohort and the 20 cases included in the independent cohort (EORTC = as described by the European Organization for Research and Treatment of Cancer-Invasive Fungal Infections Cooperative Group/National Institute of Allergy and Infectious Diseases Mycosis Study Group, HCT = hematopoietic stem cell transplantation, MDS = myelodysplastic syndrome, AML = acute myeloid leukemia, MF = myelofibrosis, ALL = acute lymphoblastic leukemia, CML = chronic myeloid leukemia).

| **Primary cohort** | | | | | | | | | | |
| --- | --- | --- | --- | --- | --- | --- | --- | --- | --- | --- |
| **Case** | **Sex** | **Age** | **Underlying**  **disease** | **EORTC**  **certainty** | **Treatment** | **Previous**  **allo-HCT** | **Neutro-penia** | **High dose**  **steroids** | **T-lymphocyte**  **inhibitors** | **EORTC**  **response**  **week 6** |
| 1 | F | 45 | MDS | Probable | Caspofungin | Yes | Yes | No | Yes | Death |
| 2 | M | 53 | AML | Probable | Posaconazole  VS voriconazole | Yes | Yes | No | Yes | Complete |
| 3 | M | 68 | AML | Probable | Posaconazole  VS voriconazole | No | No | No | Yes | Complete |
| 4 | M | 54 | AML | Probable | Caspofungin | No | Yes | No | Yes | Partial |
| 5 | M | 37 | ALL | Probable | Voriconazole | Yes | No | No | No | Death |
| 6 | M | 58 | AML | Proven | voriconazole | No | No | No | Yes | Partial |
| 7 | F | 35 | MF | Proven | AmphotericinB | No | Yes | No | Yes | Complete |
| 8 | F | 81 | AML | Proven | Voriconazole | No | No | No | No | Partial |
| 9 | M | 37 | Lymphoma | Probable | Voriconazole | Yes | No | No | No | Partial |
| 10 | F | 54 | AML | Probable | AmphotericinB | No | No | No | Yes | Partial |
| 11 | M | 34 | Lymphoma | Probable | Voriconazole | Yes | No | No | Yes | Partial |
| 12 | M | 44 | Lymphoma | Proven | Posaconazole | Yes | Yes | Yes | No | Death |
| 13 | M | 56 | AML | Probable | Voriconazole | No | No | No | Yes | Complete |
| 14 | M | 61 | ALL | Probable | Posaconazole | No | Yes | No | Yes | Progression |
| 15 | M | 71 | AML | Probable | AmphotericinB | No | Yes | No | Yes | Death |
| 16 | M | 68 | AML | Probable | Caspofungin | No | Yes | No | Yes | Death |
| 17 | F | 54 | AML | Probable | Olorofim | Yes | No | No | Yes | Death |
| 18 | F | 49 | AML | Probable | Voriconazole | Yes | Yes | No | No | Death |
| 19 | M | 67 | AML | Probable | Voriconazole | No | Yes | No | Yes | Partial |
| 20 | F | 28 | Lymphoma | Probable | AmphotericinB | No | No | Yes | Yes | Death |
| 21 | F | 53 | Lymphoma | Probable | AmphotericinB | Yes | No | No | No | Death |
| 22 | F | 60 | Lymphoma | Probable | Voriconazole | No | Yes | No | Yes | Unevaluable |
| 23 | F | 61 | CML | Probable | Voriconazole | Yes | No | No | Yes | Unevaluable |
| 24 | F | 56 | AML | Probable | Voriconazole | No | Yes | Yes | Yes | Death |
| 25 | F | 66 | AML | Probable | Voriconazole | No | Yes | No | No | Partial |
| 26 | M | 56 | AML | Probable | Posaconazole | No | Yes | No | Yes | Partial |
| 27 | F | 53 | AML | Probable | Voriconazole | No | No | Yes | No | Partial |
| 28 | M | 60 | AML | Probable | Olorofim | Yes | No | Yes | No | Partial |
| 29 | M | 40 | MDS | Proven | Voriconazole | Yes | Yes | No | Yes | Unevaluable |
| 30 | M | 71 | MM | Probable | Voriconazole | No | Yes | No | Yes | Unevaluable |
| 31 | M | 73 | AML | Probable | Voriconazole | No | Yes | No | No | Partial |
| 32 | M | 61 | AML | Probable | Voriconazole | No | Yes | No | Yes | Progression |
| 33 | F | 64 | Lymphoma | Probable | Voriconazole | No | No | No | Yes | Unevaluable |
| **Independent cohort** | | | | | | | | | | |
| **Case** | **Sex** | **Age** | **Underlying**  **disease** | **EORTC**  **certainty** | **Treatment** | **Previous**  **allo-HCT** | **Neutropenia** | **High dose steroids** | **T-lymphocyte inhibitors** | **EORTC response week 6** |
| 2-1 | M | 39 | MDS | Probable | Posaconazole | Yes | Yes | Yes | No | Stable |
| 2-2 | F | 66 | AML | Prove | Voriconazole | No | Yes | No | No | Partial |
| 2-3 | F | 34 | AML | Probable | AmphotericinB | No | Yes | No | No | Death |
| 2-4 | M | 34 | ALL | Probable | Voriconazole | No | Yes | Yes | No | Stable |
| 2-5 | F | 66 | ALL | Probable | Voriconazole | No | Yes | Yes | Yes | Unevaluable |
| 2-6 | F | 47 | AML | Probable | Voriconazole | Yes | Yes | No | No | Partial |
| 2-7 | M | 62 | AML | Probable | Voriconazole | Yes | Yes | Yes | No | Partial |
| 2-8 | F | 76 | AML | Probable | Voriconazole | No | Yes | No | Yes | Unevaluable |
| 2-9 | M | 76 | Other | Probable | AmphotericinB | No | Yes | No | No | Complete |
| 2-10 | M | 55 | ALL | Probable | Posaconazole | No | Yes | Yes | Yes | Unevaluable |
| 2-11 | F | 69 | AML | Proven | Olorofim | No | Yes | No | Yes | Partial |
| 2-12 | M | 27 | Other | Proven | Isavuconazole | No | Yes | Yes | No | Death |
| 2-13 | M | 69 | MDS | Probable | Isavuconazole | No | No | Yes | No | Partial |
| 2-14 | M | 29 | AML | Probable | Voriconazole | No | Yes | No | No | Partial |
| 2-15 | F | 44 | Other | Probable | Voriconazole | Yes | No | Yes | No | Partial |
| 2-16 | F | 23 | Other | Probable | Voriconazole  (+/- SCY-078) | Yes | Yes | No | No | Complete |
| 2-17 | M | 58 | ALL | Probable | Fosmanogepix | No | Yes | No | No | Death |
| 2-18 | M | 70 | MDS | Probable | Voriconazole  (+/- SCY-078) | No | Yes | No | No | Complete |
| 2-19 | F | 70 | AML | Probable | Voriconazole  (+/- SCY-078) | No | Yes | Yes | No | Complete |
| 2-20 | M | 71 | Other | Probable | Posaconazole | Yes | No | Yes | No | Unevaluable |

Supplementary Table 2

Overview of the main characteristics of and differences between the discovery cohort and the independent cohort, including characteristics of all cases and all controls (ALL = acute lymphoblastic leukemia, AML = acute myeloid leukemia, MDS = myelodysplastic syndrome, other = hematologic malignancy other than ALL, AML or MDS, EORTC = as described by the European Organization for Research and Treatment of Cancer-Invasive Fungal Infections Cooperative Group/National Institute of Allergy and Infectious Diseases Mycosis Study Group, IA = invasive aspergillosis, allo-HCT = allogeneic stem cell transplantation, high dose steroids = ≥0.3 mg/kg corticosteroids for ≥3 weeks).

|  |  | **Discovery**  **cohort**  n = 99 | **Independent  cohort**  n = 40 |  |
| --- | --- | --- | --- | --- |
| **Characteristic** |  | **N (%)** | **N %)** | **p-value** |
| Median age |  | 60.0 years | 60.1 years | 0.23 |
| Female sex |  | 42 (42.4) | 17 (42.5) | 0.99 |
| Underlying disease |  |  |  |  |
|  | ALL | 8 (8.1) | 11 (27.5) | *0.0025* |
|  | AML | 57 (57.6) | 21 (52.5) | 0.59 |
|  | MDS | 10 (10.1) | 3 (7.5) | 0.63 |
|  | Other | 24 (24.2) | 5 (12.5) | 0.12 |
| EORTC certainty |  |  |  |  |
|  | Proven IA | 5 (5.1) | 3 (7.5) | 0.57 |
|  | Probable IA | 28 (28.3) | 17 (42.5) | 0.1 |
|  | No IA | 66 (66.7) | 20 (50.0) | 0.067 |
| Previous allo-HCT |  | 26 (26.3) | 12 (30.0) | 0.65 |
| Neutropenia |  | 35 (35.4) | 34 (85.0) | *<0.001* |
| High dose steroids |  | 12 (12.1) | 17 (42.5) | *<0.001* |
| T-lymphocyte inhibitors |  | 38 (38.4) | 8 (20.0) | *0.037* |
| Active GvHD |  | 2 (2.0) | 6 (15.0) | *0.0029* |
